# Determination of the volume of coronary microemboli needed for reproducible visualization of microinfarcts on contrast enhanced MDCT and MRI

**DOI:** 10.1186/1532-429X-14-S1-M14

**Published:** 2012-02-01

**Authors:** Loi Do, Mark Wilson, Steven W Hetts, Carol Stillson, Sammir M Sullivan, Maythem Saeed

**Affiliations:** 1 Radiology and Biomedical Imaging, University of California San Francisco, San Francisco, CA, USA

## Summary

The purpose of this study was to 1) determine the threshold volume of coronary microemboli (<120μm) needed for reproducible visualization of microinfarcts on contrast enhanced MDCT and MRI and 2) assess the impact of microemboli volumes on myocardial perfusion and left ventricular (LV) function.

## Background

Microembolization of the coronary arteries is known to occur spontaneously as a result of erosion or rupture of atherosclerotic plaque and iatrogenically during percutaneous coronary interventions (PCI).

## Methods

Under X-ray guidance, a 3F catheter was placed selectively in LAD coronary artery of pigs (n=18). The animals received either 16mm^3^ or 32mm^3^ volumes of 40-120μm Embosphere®, microemboli (Biosphere Med, Rockland, MA) were selectively delivered in the LAD coronary artery to produce microinfarcts. The microemboli used were in the same size as those that pass through the pores of distal filtration devices and the volumes found at autopsy.

On day 3, both MDCT (64-slice) and MRI (1.5T) were performed using CT iohexol (350mg/ml x 2) and MR Gd-DTPA (0.1mmol/kg x 2) contrast media. The images were used for assessing LV function, perfusion and viability (visualization of speckled microinfarcts), respectively. A semi-automatic threshold method was used to measure enhanced speckled microinfarcts. Histology was used to confirm the presence of microvascular obstruction and microinfarcts.

## Results

Contrast enhanced speckled microinfarcts were visible by the naked eye on MDCT and MRI in all animals that received 32mm^3^ of microemboli volume , but only in 2/3 of the animals that received 16mm^3^ volume. The semi-automatic threshold method provided measurements of microinfarct sizes in animals that received 16mm^3^ and 32mm^3^ volume. The contrast between microinfarcts and viable myocardium after administration of contrast media was greater on MRI than MDCT. Regional perfusion and radial strain were impaired in the territory subtended by the LAD compared with remote myocardium. Histology showed evidence of necrosis, apoptosis, inflammation, edema and microvascular obstruction in all animals.

## Conclusions

A 32mm^3^ volume of microemboli produces reproducible visualization of speckled microinfarct on contrast enhanced MDCT or MRI. At the volumes of microemboli used, the semi-automatic threshold method can measure microinfarcts. A threshold volume of microemboli for visualization of microinfarct has been established. The clinical implications of this coronary interventional MR and MDCT study are that these imaging techniques: 1) can be used interchangeably for measuring microinfarct size, regional perfusion and LV function after PCI, and 2) may help in testing the effectiveness of current and new distal filtration devices used to protect myocardium from microembolization.

## Funding

University of California Department of Radiology and Biomedical Imaging.

